# Mechanical Evaluation of Polymer Composite Hip Protectors

**DOI:** 10.1155/2010/431591

**Published:** 2010-08-12

**Authors:** Jose Daniel Diniz Melo, Ayrles S. Gonçalves Barbosa, Ricardo Oliveira Guerra

**Affiliations:** ^1^Department of Materials Engineering, The Federal University of Rio Grande do Norte, 59072-970 Natal, RN, Brazil; ^2^Department of Physical Therapy, The Federal University of Rio Grande do Norte, 59072-970 Natal, RN, Brazil

## Abstract

Hip fractures often result in serious health implications, particularly in the geriatric population, and have been related to long-term morbidity and death. In most cases, these fractures are caused by impact loads in the area of the greater trochanter, which are produced in a fall. This work is aimed at developing hip protectors using composite materials and evaluating their effectiveness in preventing hip fractures under high impact energy (120 J). The hip protectors were developed with an inner layer of energy absorbing soft material and an outer rigid shell of fiberglass-reinforced polymer composite. According to the experimental results, all tested configurations proved to be effective at reducing the impact load to below the average fracture threshold of proximal femur. Furthermore, an addition of Ethylene Vinyl Acetate (EVA) to the impacted area of the composite shell proved to be beneficial to increase impact strength of the hip protectors. Thus, composite hip protectors proved to be a viable alternative for a mechanically efficient and cost-effective solution to prevent hip fractures.

## 1. Introduction

Hip fracture in the elderly population is an important worldwide health concern. It has been associated with high morbidity and increased risk of death, with up to 20% of the elderly dying within six months of the fracture [1]. Previous studies suggested that the impact force applied on the area of the greater trochanter in sideways falls is the main cause of hip fractures [2–4]. Although the impact force applied to the greater trochanter area during a fall may be sufficient to cause hip fractures in healthy young individuals [5], elderly people are in general more prone to hip fractures due to reduced upper body strength, coordination, and speed [1]. Additionally, the probability of fracture increases if bones are weakened by diseases such as osteoporosis [5].

Hip protectors are devices designed to reduce the risk of fall-related hip fractures in individuals, particularly for those people who have compromised bone strength. They have been proven effective in decreasing the risk of hip fracture in elderly nursing home residents [6]. Various configurations of hip protectors have been studied to reduce the probability of hip fracture in the event of a fall [1, 7, 8]. Energy-absorbing hip protectors are designed to attenuate impact forces by means of a soft shock-absorbing material, while energy-shunting devices distribute impact loads away from the greater trochanter to the surrounding soft tissues. Previous investigation indicated that the force attenuation capacity of hard hip protectors may be significantly better than the soft ones [9]. Some devices combine both energy shunting and energy absorption into one product [1, 4, 7, 8]. For these devices, composite materials may offer many advantages when compared to previous designs which use more conventional isotropic materials.

Composite materials have been largely used in aerospace and aircraft industries in particular due to their high specific mechanical properties such as stiffness and strength, design flexibility and reduced weight. With composite materials, structures can be designed using a variety of reinforcement types and orientations, diverse matrix materials, and layup sequences to achieve superior properties. Properties of composite materials can be tailored to provide specific energy absorption capabilities superior to those of metals and polymers. Previous investigations have indicated that the energy absorption mechanisms in composite materials are more complex than those observed in conventional materials and include matrix cracking, delamination, and fiber breakage [10, 11]. Therefore, the use of composites is an appealing option to substitute more traditional materials in applications where superior impact resistance is desired. Composites have been proven advantageous for hip prosthesis in terms of long-term stability because when compared to titanium alloys they produce less stress shielding which may lead to bone resorption, as demonstrated in a previous study [12]. In the case of orthesis for hip joint protection, the use of composite materials allows the manufacture of custom-made devices with superior properties according to the biotype of the user.

In the present paper, hip protectors were fabricated with an outer rigid shell of fiberglass reinforced polymer composite and an inner layer of energy absorbing material and then tested under impact load. Samples were produced with three different configurations, and their effectiveness in providing protection was assessed.

## 2. Experimental

### 2.1. Materials and Test Specimens

**Figure 1:**
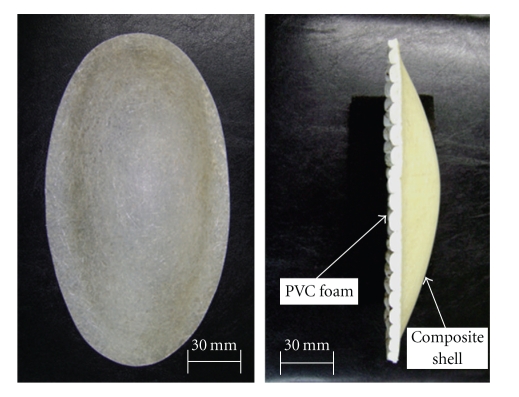
Composite hip protectors.

The structure of the hip protectors consisted of a polymer composite rigid shell and a inner layer of soft material designed for energy-shunting and energy-absorption, respectively. The outer shell was made of glass fiber-reinforced polyester composite to resist the impact force, while the inner layer was made of Poly Vinyl Chloride (PVC) foam material for energy-absorption and improved comfort (Figure 1). Comfort is a key issue to improve acceptability and adherence with use of hip protectors [13].

For the fabrication of the outer shell, orthophthalic polyester resin (Ara Ashland AZ 4.5) was prepared with 1 wt. % MEKP catalyst and glass fiber chopped strand mat (Owens Corning M710B, 450 g/m²) was used as reinforcement. Chopped strand mats (CSM) are used as reinforcements for many hand layup applications. They are very economical, provide good stiffness and conform easily to highly contoured molds. In addition, with CSM laminae a transversely isotropic material may be obtained.

In some of the protectors produced, EVA particles were also added to the polyester matrix with the intent of improving the impact resistance of the composite. Particle-size distribution of EVA was determined with a Viatest Mechanical Sieve Shaker by using a series of sieves for a shaking period of 20 min. The percentage (by mass) of particles passing each sieve size was 100% for sieve no. 30 (0.595 mm), 74% for sieve No. 50 (0.297 mm) and 13% for sieve No. 100 (0.150 mm).

The outer composite shell was manufactured using vacuum-assisted hand layup process. A layer of polyester gel coat was applied on the mold previously prepared with wax mold release. For the composites in which EVA was added, gel coat was prepared with EVA particles content of 10 wt. %. This was found to be the maximum particle content to permit a proper resin flow on the mold. After the gel coat was applied, glass fiber chopped strand mat was pre-impregnated with resin and arranged manually in the mold. The process was repeated according to the number of layers of each protector. Brushes and rollers were used to remove excess resin, porosities and air bubbles and to improve consolidation.

After the layup process was complete, the composite material was degassed for 30 min using a vacuum bag and allowed to cure for 24 h at ambient temperature. Excess material at the edges was trimmed off using a diamond abrasive saw. All specimen edges were sanded for better finish. The final dimensions were 190 mm (length), 97 mm (width) and 30 mm (height). These optimized dimensions were defined based on a previous study published in the literature [7]. Three shell configurations were fabricated for the impact tests: the first configuration used two layers of glass fiber chopped strand mat, the second configuration used three layers of glass fiber chopped strand mat and the third configuration used one layer of glass fiber chopped strand mat and gel coat with EVA particles content of 10 wt. %. Thus, the measured thicknesses of the composite shells varied according to the configuration used: 1.2 mm for two layers of chopped strand mat, 1.6 mm for three layers and 0.6 mm for shells with one layer of chopped strand mat and EVA 10 wt. %. After the outer shell was fabricated, the inner layer of PVC foam material (10 mm thickness) was bonded to the shell using polyester resin.

### 2.2. Testing Apparatus and Calibration Procedure

**Figure 2:**
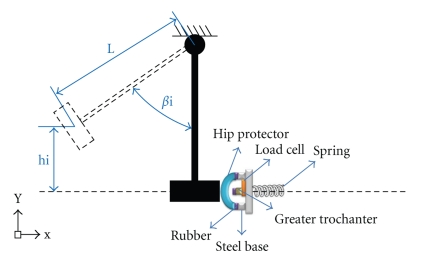
Schematic of the impact test equipment.

**Figure 3:**
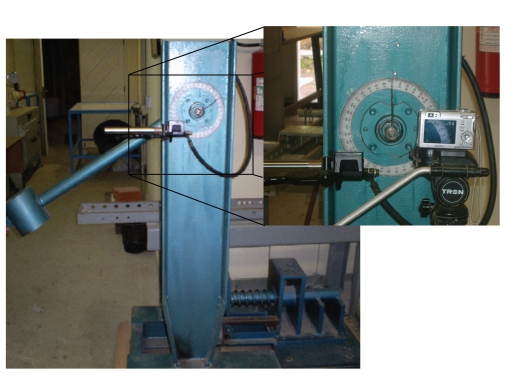
Impact test equipment.

A custom-made pendulum impact machine equipped with a load cell was developed for the impact tests (Figures 2 and 3). The apparatus was equipped with a pendulum holding and releasing mechanism and a pointer and dial mechanism for indicating the initial height of rise of the pendulum. The pendulum was designed to deliver impact energy of 120 J. Similar impact energies have been used in other studies reported in the literature [1, 7, 14].

Custom-made hip-shaped aluminum parts were produced by a sand casting process similar to those produced in a previous study published in the literature [1]. The casting mold was formed using a human femur as a pattern obtained from an adult female cadaver. The artificial greater trochanter part was mounted on a load cell (FLINTEC model BK2, max load 19.61 kN) using a center bolt. The load cell was bolted to a steel basis mounted on a spring (k = 77.5 ± 3.9 kN/m) to simulate pelvic compliance [7] and connected to a HBM Spider 8 PC-based data acquisition system to measure the impact forces. A 20 mm thick layer of elastomeric material was placed on top of the surrogate greater trochanter and also on the base for mounting the hip protectors to simulate the properties of the soft tissues covering the greater trochanter area.

The effective mass and effective length of the pendulum were determined to be 24.66 kg and 0.8366 m, respectively, as per ASTM D6110 [15]. The striking mass was made of steel and the center of percussion was determined experimentally from the period of motion of small amplitude oscillations of the pendulum according to ASTM D6110.

The testing machine was calibrated before the impact tests to account for windage and friction losses. The procedure for the calculation of windage and friction correction was based on the assumption that the losses are proportional to the angle through which these loss torques are applied to the pendulum as stated in annex A.1 of ASTM D6110. The energy correction for windage and friction was determined with one complete swing of the pendulum without a specimen in the testing device. Then, the correction was considered between the release position and the free hanging position where impact occurs. A height of rise of the pendulum was defined as 0.5077 m to deliver an impact energy of 120 J, considering windage and friction. This elevation of the striking mass is obtained by raising the pendulum to an angle 67° with respect to the free hanging position. The speed of the striking mass at the moment of impact was approximately 3.12 m/s as determined by the conservation of energy equation. This impact velocity is within the typical range reported in the literature (3.17 ± 0.47 m/s) [16].

### 2.3. Testing Procedure

The first set of tests was conducted without the hip protectors. First the impact load was applied directly on the custom made artificial greater trochanter aluminum part mounted on the load cell. The pendulum was raised to an angle 67° with respect to the free hanging position and secured in the release mechanism. Then, the pendulum was released allowing the striking mass to impact the hip-shaped aluminum cast part. The impact load was measured and the aluminum part was replaced for every repetition.

For the second type of test, the surrogate greater trochanter was covered by the 20 mm thick layer of elastomeric material which simulates the soft tissues. The procedure for the impact test was repeated and the impact load measured. The rubber soft tissue was replaced after each impact test. This procedure allowed the determination of the impact force attenuation produced by the elastomeric layer.

Following the determination of the impact forces with and without the elastomeric layer, impact tests were conducted on the hip protectors. First, the artificial greater trochanter aluminum part was mounted on the load cell and covered by the 20 mm thick layer of elastomeric material. The base for mounting the hip protectors was also covered by the same material. Then, the hip protector was positioned and centered on the base so that the center of percussion of the pendulum would strike it at the point of maximum height. The pendulum was raised to an angle 67° with respect to the free hanging position and secured in the release mechanism. The pendulum was then released and the striking mass impacted the specimen.

All hip protectors were weighed before the impact tests. Weight is an important design parameter because low weight improves user compliance. For all procedures described, five individual impact tests were conducted to ensure repeatability for a particular sample.

## 3. Results and Discussion

The mass of hip protectors is a very important parameter for user compliance as greater weight would certainly cause discomfort. A comparison of the mass of the protectors fabricated is given in Table 1. As it can be observed in these data, hip protectors with two layers of fiber-reinforced composite or one composite layer with EVA added resulted in very similar weights. However, for hip protectors with three layers of fiber-reinforced composite, the weight doubled. In this case, although only one extracomposite layer was added, the resin removal during vacuum bagging was not as efficient as in specimens with one or two composite layers which resulted in extraweight. The short gel time for the polyester resin (approximately 5 min) was one of the factors that limited the process. However, even for these protectors the mass is considered appropriate for the intended application.

The first impact tests were conducted with the impact load being applied directly on the surrogate greater trochanter made of aluminum. In this case, an average impact load of 14.84 kN with standard deviation of 0.78 kN was registered by the load cell for the 120 J impact. When the layer of rubber was placed on top of the surrogate greater trochanter to simulate the properties of the soft tissues, the average registered load was reduced to 11.50 kN with standard deviation of 0.48 kN. Thus, the elastomeric layer was found to attenuate 22.5% of the impact force, which is within the range of experimentally determined force attenuation in trochanteric soft tissues [9, 14]. Impact force attenuation and energy absorption in soft tissues have been shown in previous research as dependent upon tissue thickness. Thus, as tissue thickness increases, peak force decreases and tissue energy absorption increases [14].

**Figure 4:**
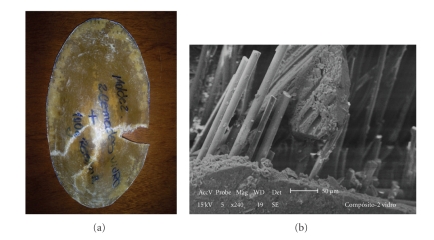
Macroscopic and microscopic (SEM) details of the fracture (2 layers).

After the impact tests were conducted without hip protectors, the fabricated composite hip protectors were impact tested. Protectors with two fiber-reinforced layers fractured with the applied impact (Figure 4). As the fracture occurred and the protector deformed, the internal surface of the protector contacted the elastomeric layer of surrogate soft tissues. The average impact force registered by the load cell was 0.33 kN with standard deviation of 0.02 kN.

Microscopic analysis of the fractured specimens suggests that the fracture initiated in the region impacted by the pendulum and propagated in various directions to the contour of the specimen. The SEM fractograph (Figure 4) shows microscopic details of the fractured region. Due to the brittle nature of the glass fibers and polyester matrix, the material does not undergo plastic deformation. The shock wave produced by the impact caused matrix fragmentation and, consequently, fiber/matrix debonding, thus exposing the fibers. These were the main mechanisms of impact energy dissipation for this fiber-reinforced composite shell. Although the load registered at the load cell was safe as compared to the threshold value of 2.5 kN [1], the failure of this protector would certainly have caused discomfort to users, even though it would be able to prevent a hip fracture. In this case, the inner PVC foam layer would protect the user from cuts as the outer shell failed, in addition to its role of energy-absorption.

**Figure 5:**
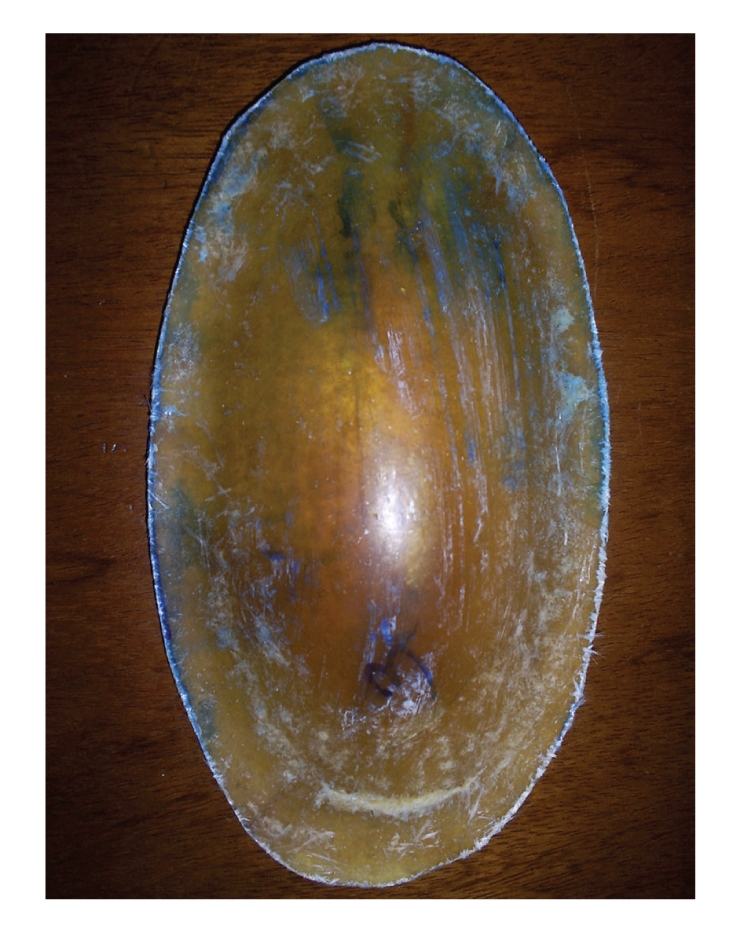
Composite hip protector with three layers on the reinforced shell.

Hip protectors with three layers of glass fiber chopped strand mat maintained their structural integrity under the applied impact load. Fracture was not observed in these specimens and no impact force was registered by the load cell (Figure 5). Thus, when these hip protectors were used, no impact force was applied to the artificial greater trochanter. In this case, the hip protectors were able to shunt the impact energy away from the greater trochanter area and distribute it to the adjacent tissues.

**Figure 6:**
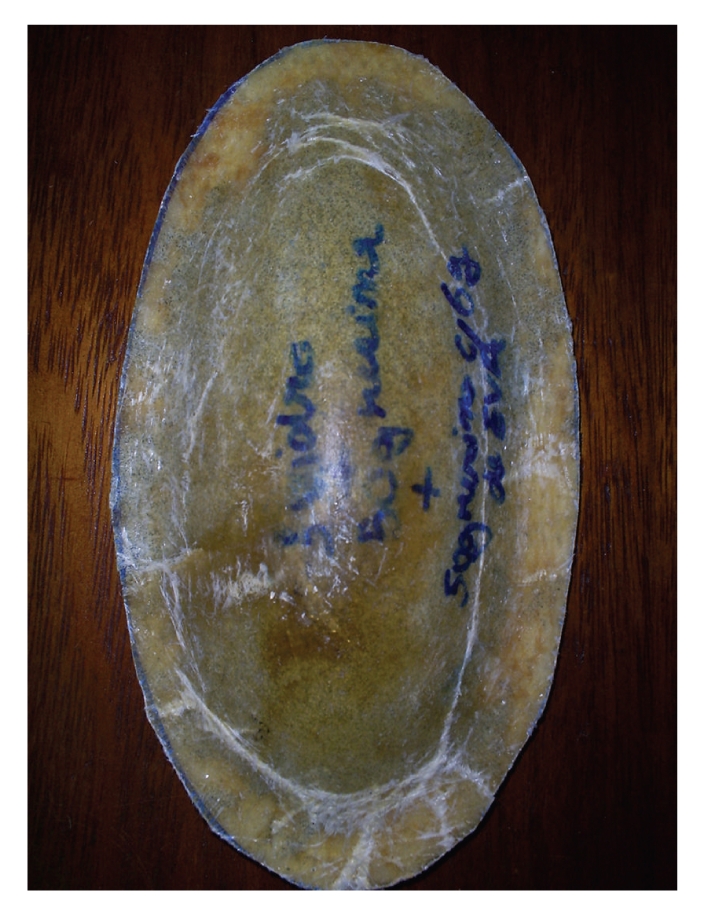
Hip protectors fabricated with one layer of glass fiber chopped strand mat and gel coat with EVA particles content of 10 wt. %, after impact load.

As in the case of hip protectors fabricated with two layers of glass fiber chopped strand mat on the outer shell, those produced with one layer of fiber chopped strand mat and having EVA particles content of 10 wt. % on the gel coat failed with the impact load (Figure 6). These specimens had only one layer of fiber-reinforced polymer and therefore were not able to resist the impact load. The strength was further reduced by the presence of the elastomeric filler [17]. As these protectors failed, an average impact load of 1.23 kN with a standard deviation of 0.16 kN was registered by the load cell. However, the fracture aspect of this tested configuration was different. In this case, although fracture occurred, pieces of the fractured specimen remained attached to the protectors. The failure occurred in the region close to the contour of the specimen, along a path where there is a remarkable change in surface curvature of the outer shell (Figure 6). However, the area impacted by the pendulum did not fail under impact as it occurred with the specimens with two layers, even though this specimen had only one layer of polymer composite. Therefore, the elastomeric filler improved the impact resistance of the outer shell. This observation is in agreement with results previously published in the literature which indicated that the elastomeric properties of the EVA particles can effectively improve the impact properties of the polyester matrix [17].

**Figure 7:**
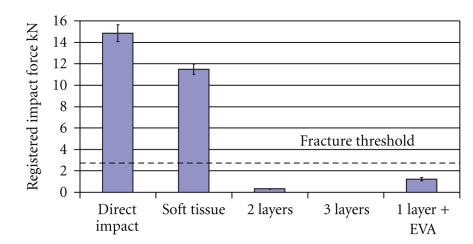
Impact tests data showing the impact on the surrogate greater trochanter (direct impact), the impact on the surrogate greater trochanter covered by the soft tissue, and the impact on the three hip protectors configurations. Error bars represent plus or minus one standard deviation.

The results of all impact tests conducted in this investigation are summarized in Figure 7. In addition, the line of fracture threshold of 2.5 kN is included for comparison purposes. It can be observed that all hip protectors studied in this work were capable of reducing the impact load to the safe range even though some of the configurations tested failed under impact. The use of EVA particles proved to be a viable option to improve the impact properties of the material and opens the possibility of producing hip protectors to meet the requirements for mechanical properties at lower cost. However, among the configurations evaluated in this work, only the composite hip protectors with three layers of glass fiber mat were proven suitable for the intended application.

## 4. Conclusions

In this investigation, polymer composite hip protectors were fabricated and their effectiveness in preventing hip fractures was evaluated through impact tests. The hip protectors were constructed of an outer glass fiber/polyester composite shell and an inner layer of PVC foam for energy-shunting and energy-absorption, respectively. Three configurations were tested according to the composition of the outer shell: two layers of polymer composite, three layers of polymer composite and one layer of polymer composite with the addition of EVA particles. The weight of all protectors was under 105 g which is considered acceptable to provide user comfort. All hip protectors were found capable of preventing the impact force from reaching the threshold value of 2.5 kN when subjected to an impact energy of 120 J. However, among all specimens tested, only those with three layers of polymer composite in the outer shell did not fail under the applied impact load. With these protectors, the load was completely distributed to the surrogate soft tissues surrounding the greater trochanter. Experimental data indicates that the impact resistance of the hip protectors can be improved by the addition of EVA particles. In summary, according to the experimental data obtained in this investigation, the use of composite hip protectors may be an efficient alternative to provide protection to hip fracture particularly for the elderly population. The use of composite materials offers the advantages of low weight and design flexibility, which provides the possibility of producing custom made hip protectors according to the specific user needs.

## Figures and Tables

**Table 1:** Mass of the hip protectors.

Hip protector configuration	mass (g)	
	x	s
2 layers of FR composite	65	3
3 layers of FR composite	102	3
1 layer of FR composite + EVA 10 wt. %	60	5

x = arithmetic mean of the set of observations.

s = estimated standard deviation.
